# Yersinia pseudotuberculosis-Associated Myositis

**DOI:** 10.7759/cureus.73399

**Published:** 2024-11-10

**Authors:** Koji Yokoyama, Mitsukazu Mamada

**Affiliations:** 1 Department of Pediatrics, Japanese Red Cross Wakayama Medical Center, Wakayama, JPN

**Keywords:** antibiotics, myositis, pediatric reactive arthritis, treatment, yersinia pseudotuberculosis infection

## Abstract

*Yersinia pseudotuberculosis* (*Y. pstb.*) is a Gram-negative pathogen associated with gastrointestinal infections, such as enterocolitis. While complications like reactive arthritis can occur, progression to myositis is extremely rare. This report describes a five-year-old boy who developed myositis following a *Y. pstb.* infection. The patient presented with a five-day history of abdominal pain, fever, and leg pain that progressed to muscle weakness, which affected his ability to walk. Laboratory results showed elevated inflammatory markers, and stool cultures confirmed *Y. pstb.* infection. Magnetic resonance imaging (MRI) examination revealed muscle inflammation consistent with myositis. The patient improved rapidly with conservative treatment (rest and acetaminophen) and did not require antibiotics. Thirteen years later, he remained asymptomatic. This case highlights the rare complication of myositis following *Y. pstb.* infection. Potential mechanisms include postinfectious immune-mediated myositis. Clinicians should be aware of this possibility, and conservative treatment may suffice for recovery.

## Introduction

*Yersinia pseudotuberculosis* (*Y. pstb.*) is a Gram-negative zoonotic pathogen [1]. Although infections of *Y. ptsb. *occur less frequently than those of its relative *Yersinia enterocolitica* (*Y. ent.*), they are commonly observed in children [1]. *Y. ptsb.* infection presents with five primary clinical syndromes, namely, acute gastroenteritis, mesenteric lymphadenitis, erythema nodosum, septicemia, and reactive arthritis [2,3]. In pediatric cases of *Y. pstb.*, differential diagnosis is essential, given that common symptoms such as rash and abdominal pain can closely mimic those of other conditions [4]. Among these five primary symptoms, reactive arthritis has been reported infrequently in the literature, and progression to myositis remains exceedingly rare [2,5]. This case report details the presentation of a pediatric male with *Y. pstb. *infection that progressed to myositis and discusses the potential mechanisms and clinical implications.

## Case presentation

**Figure 1:**
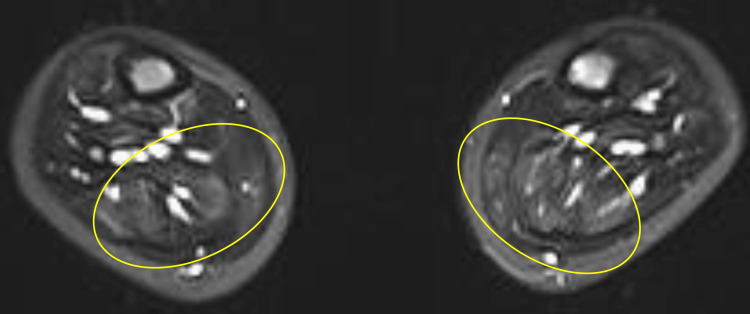
The transverse section of the MRI scan using STIR shows multiple high-signal intensities, predominantly in the lower limbs. The yellow rings indicate areas of signal change STIR: Short tau inversion recovery

**Figure 2:**
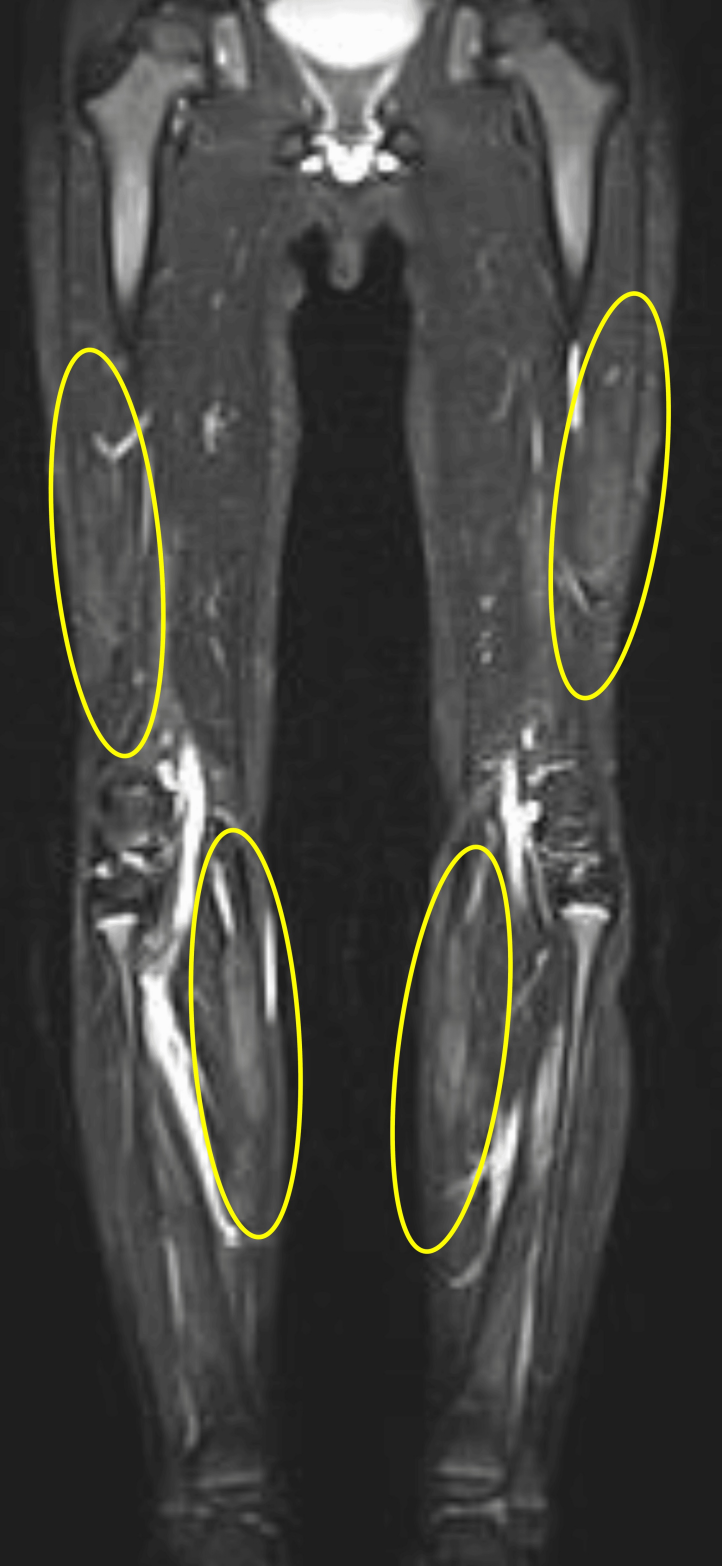
The coronal section of the MRI scan using STIR MRI: Magnetic resonance imaging; STIR: short tau inversion recovery

A five-year-old boy weighing 12.3 kg presented with a five-day history of abdominal and bilateral calf and thigh pain, fever, and diarrhea. Two days after the onset of gastrointestinal symptoms, the patient developed severe bilateral gastrocnemius pain and weakness, which significantly impaired his ability to ambulate. He and his guardians denied any history of raw meat consumption, well water ingestion, swimming in lakes or ponds, animal contact, or recent international travel. He had no notable medical history, kept no pets, had no known allergies, had been previously vaccinated, and had no relevant family medical history. Upon admission, the patient presented with blood pressure of 90/50 mmHg, a heart rate of 114 beats per minute, a respiratory rate of 18 breaths per minute, a tympanic temperature of 40.6°C, and oxygen saturation of 100% on room air. His abdomen was flat and soft but tender to palpation, and he did not exhibit any joint-related symptoms. Laboratory evaluation revealed elevated inflammatory markers, including C-reactive protein (5.41 mg/dL), and an elevated erythrocyte sedimentation rate (59 mm/1 hour). Infection with *Y. pstb.* was confirmed through positive stool cultures. On the 23rd day of illness, the *Y. pstb.* agglutinin titer (serotype 4b) had significantly increased to 1:320 (reference range: <1:160), while the anti-*Y. pstb.*-derived mitogen (YPM) antibody titer had markedly increased to 11.8 (reference range: <2.5). Serum agglutinin titers and anti-YPM antibodies were measured in accordance with previously described methods [6,7]. The creatine phosphokinase level, the aldolase level, antinuclear antibody test results, anti-myositis antibodies, and the lactate/pyruvate ratio were all within normal limits. Blood culture, stool viral cultures, rapid influenza antigen tests, and tests for serum antibodies against echovirus and coxsackievirus were negative. Abdominal X-ray and ultrasound examinations revealed no abnormalities. Magnetic resonance imaging (MRI) of the lower extremities demonstrated inflammation consistent with myositis, which was particularly prominent in the lower limb muscles [8]. MRI showed an increase in short tau inversion recovery (STIR) signal intensity (Figures 1-2).

Remarkably, the patient’s symptoms resolved within a few days with conservative management consisting of rest and acetaminophen for analgesia, without the use of antibiotics. A muscle biopsy was not planned because his muscle pain had improved after admission. Follow-up MRI on the 23rd day of illness confirmed that the inflammatory changes in the affected muscles were completely resolved. Thirteen years have passed, and since then, symptoms have not recurred.

## Discussion

This case highlights an unusual manifestation of *Y. pstb.* infection, namely, its progression to myositis in a previously healthy pediatric patient. Although *Y. pstb.* infection typically presents with gastrointestinal involvement, clinicians should be aware of rare systemic complications such as myositis. The successful resolution of the patient’s symptoms with conservative treatment, without the need for antimicrobial therapy, suggests a self-limiting inflammatory process, although the exact pathogenesis remains unclear. Several potential mechanisms may explain how *Y. pstb.* infection can lead to myositis in pediatric patients. First is a superantigen-mediated immune response. *Y. pstb.* produces a superantigenic toxin, the YPM antibody, which can trigger an exaggerated immune response. This hyperactivation of the immune system may lead to systemic inflammation, including in muscle tissue [9]. Although this case tested positive for YPM antibodies, neither antibiotics nor immunomodulatory treatments were necessary. The clinical course of a positive case is likely influenced by the patient's overall condition, as well as the bacterial load and strain involved. Second is the direct bacterial invasion. Bacteremia resulting from gastrointestinal translocation of bacteria can lead to seeding in distant tissues, including muscles [10]. Although rare, direct infection of muscle tissue could lead to localized inflammation. Notably, there have been reports of *Y. ent.* directly causing polymyositis with involvement of the gastrocnemius muscle [11]. In our case, symptoms improved with rest and analgesics without the use of antibiotics, and direct invasion of muscles by *Y. pstb.* is considered unlikely. Third is postinfectious immune-mediated myositis. Similar to reactive arthritis, postinfectious myositis could occur as a result of an aberrant immune response following the initial gastrointestinal infection. This immune-mediated mechanism may be triggered by molecular mimicry, where bacterial antigens resemble host muscle tissue proteins [12,13]. Persistent *Y. pstb.* infections in joints have been detected in some patients, indicating the bacterium’s potential to contribute to chronic arthritis in genetically predisposed individuals, such as those with the HLA-B27 gene​ [13]. This case suggests that myositis associated with *Yersinia* infection may share pathological features with reactive arthritis, particularly given the rapid improvement observed within 2-4 weeks with rest and the use of analgesics. This short duration and favorable response to conservative treatment are consistent with the self-limiting nature of reactive inflammation triggered by infection [14,15]. Although myositis is a rare manifestation of *Yersinia* infection, the clinical course of this case aligns with the mechanism of postinfectious reactive inflammation, where the immune response to the pathogen results in musculoskeletal involvement without the need for prolonged intervention. Genetic testing, including HLA typing, was not performed in this case. 

## Conclusions

This case of *Y. pstb.*-associated myositis in a pediatric patient underscores the importance of recognizing uncommon systemic complications associated with *Y. pstb.* infections. To our knowledge, this is the first report confirming myositis associated with this pathogen based on MRI findings. Although rare, clinicians should be aware that musculoskeletal symptoms such as myositis may develop in children with *Y. pstb.* infections, particularly those with prolonged or severe systemic inflammation. Conservative measures, including rest and analgesia with acetaminophen, may be sufficient to achieve a favorable outcome in cases without severe systemic involvement.

## References

[1] Brady MF, Yarrarapu SNS, Anjum F (2024). Yersinia pseudotuberculosis. StatPearls. Treasure Island.

[2] (2024). Yersinia enterocolitica and Yersinia pseudotuberculosis infections (enteritis and other llnesses). Red Book: 2021-2024 Report of the Committee on Infectious Diseases, Committee on Infectious Diseases.

[3] Shane AL, Mody RK, Crump JA (2017). 2017 Infectious Diseases Society of America clinical practice guidelines for the diagnosis and management of infectious diarrhea. Clin Infect Dis.

[4] Long C, Jones TF, Vugia DJ (2010). Yersinia pseudotuberculosis and Y. enterocolitica infections, FoodNet, 1996-2007. Emerg Infect Dis.

[5] Kaasch AJ, Dinter J, Goeser T, Plum G, Seifert H (2012). Yersinia pseudotuberculosis bloodstream infection and septic arthritis: case report and review of the literature. Infection.

[6] Abe J, Onimaru M, Matsumoto S (1997). Clinical role for a superantigen in Yersinia pseudotuberculosis infection. J Clin Invest.

[7] Nakajima H, Inoue M, Mori T, Itoh K, Arakawa E, Watanabe H (1992). Detection and identification of Yersinia pseudotuberculosis and pathogenic Yersinia enterocolitica by an improved polymerase chain reaction method. J Clin Microbiol.

[8] Albayda J, Demonceau G, Carlier PG (2022). Muscle imaging in myositis: MRI, US, and PET. Best Pract Res Clin Rheumatol.

[9] Ocho K, Iwamuro M, Hasegawa K, Hagiya H, Rai K, Yumoto T, Otsuka F (2018). Far East scarlet-like fever masquerading as adult-onset Kawasaki disease. Intern Med.

[10] Radcliffe C, Gisriel S, Niu YS, Peaper D, Delgado S, Grant M (2021). Pyomyositis and infectious myositis: a comprehensive, single-center retrospective study. Open Forum Infect Dis.

[11] Brennessel DJ, Robbins N, Hindman S (1984). Pyomyositis caused by Yersinia enterocolitica. J Clin Microbiol.

[12] Borg AA, Gray J, Dawes PT (1992). Yersinia-related arthritis in the United Kingdom. A report of 12 cases and review of the literature. Q J Med.

[13] Lucchino B, Spinelli FR, Perricone C, Valesini G, Di Franco M (2019). Reactive arthritis: current treatment challenges and future perspectives. Clin Exp Rheumatol.

[14] Vasala M, Hallanvuo S, Ruuska P, Suokas R, Siitonen A, Hakala M (2014). High frequency of reactive arthritis in adults after Yersinia pseudotuberculosis O:1 outbreak caused by contaminated grated carrots. Ann Rheum Dis.

[15] Toivanen A, Granfors K, Lahesmaa-Rantala R, Leino R, Ståhlberg T, Vuento R (1985). Pathogenesis of Yersinia-triggered reactive arthritis: immunological, microbiological and clinical aspects. Immunol Rev.

